# Induced secretion system mutation alters rhizosphere bacterial composition in *Sorghum bicolor* (L.) Moench

**DOI:** 10.1007/s00425-021-03569-5

**Published:** 2021-01-18

**Authors:** Vimal Kumar Balasubramanian, Lavanya Dampanaboina, Christopher Joseph Cobos, Ning Yuan, Zhanguo Xin, Venugopal Mendu

**Affiliations:** 1 Fiber and Biopolymer Research Institute (FBRI), Department of Plant and Soil Science, Texas Tech University, Lubbock, TX 79409 USA; 2 USDA-ARS, Lubbock, TX USA; 3 Department of Plant and Soil Science, Texas Tech University, Lubbock, 79409 USA

**Keywords:** EMS mutant, Induced secretion system, Microbiome, Red root, Rhizosphere, Root exudates

## Abstract

**Main conclusion:**

A novel inducible secretion system mutation in Sorghum named *Red root* has been identified. The mutant plant root exudes pigmented compounds that enriches Actinobacteria in its rhizosphere compared to BTx623.

**Abstract:**

Favorable plant–microbe interactions in the rhizosphere positively influence plant growth and stress tolerance. *Sorghum bicolor*, a staple biomass and food crop, has been shown to selectively recruit Gram-positive bacteria (Actinobacteria) in its rhizosphere under drought conditions to enhance stress tolerance. However, the genetic/biochemical mechanism underlying the selective enrichment of specific microbial phyla in the sorghum rhizosphere is poorly known due to the lack of available mutants with altered root secretion systems. Using a subset of sorghum ethyl methanesulfonate (EMS) mutant lines, we have isolated a novel *Red root* (*RR*) mutant with an increased accumulation and secretion of phenolic compounds in roots. Genetic analysis showed that *RR* is a single dominant mutation. We further investigated the effect of root-specific phenolic compounds on rhizosphere microbiome composition under well-watered and water-deficit conditions. The microbiome diversity analysis of the *RR* rhizosphere showed that Actinobacteria were enriched significantly under the well-watered condition but showed no significant change under the water-deficit condition. BTx623 rhizosphere showed a significant increase in Actinobacteria under the water-deficit condition. Overall, the rhizosphere of *RR* genotype retained a higher bacterial diversity and richness relative to the rhizosphere of BTx623, especially under water-deficit condition. Therefore, the *RR* mutant provides an excellent genetic resource for rhizosphere-microbiome interaction studies as well as to develop drought-tolerant lines. Identification of the *RR* gene and the molecular mechanism through which the mutant selectively enriches microbial populations in the rhizosphere will be useful in designing strategies for improving sorghum productivity and stress tolerance.

**Supplementary Information:**

The online version contains supplementary material available at 10.1007/s00425-021-03569-5.

## Introduction

The rhizosphere microbiome composition promotes plant growth by nutrient solubilization, carbon sequestration, nitrogen fixation, induction of disease resistance, and phytohormone biosynthesis in plants (Mabood et al. 2014; Smith et al. 2015). Plants manage root microbial flora population by secreting root exudates into the rhizosphere, which act as a carbon source for the underground microbiome. It was estimated that 20% of photosynthetically fixed carbon was used for producing root exudates in the plant rhizosphere (Kuzyakov and Domanski 2000). Root exudate composition is very critical in selective enrichment of microbial species around the rhizosphere (Bais et al. 2006). These exudates are rich in phenolic compounds, carbohydrates, proteins, among which flavonoids play a critical role in attracting beneficial microbes and in plant defenses against pathogens, herbivores and environmental stress (Treutter 2005). Plant roots use these biochemicals as molecular signals to attract, repel, or maintain microbial species in the rhizosphere (Tseng et al. 2009; Nelson and Sadowsky 2015). Benzoxazinoids in maize root exudate were shown to specifically enrich *Pseudomonas putida* (Neal et al. 2012), and oxylipins in tomato root exudate were shown to specifically enrich *Trichoderma harzianum* (Lombardi et al. 2018). Further, flavonoids such as catechin and coumarin are involved in microbial metabolism and niche establishment (Wang et al. 2013; Stringlis et al. 2018). Therefore, understanding the genetic and molecular mechanisms behind the root secretion system and root exudate composition is important for the fundamental understanding of plant-microbe interaction, as well as to design strategies for crop improvement.

*Sorghum bicolor* is a major C4 crop grown for food grains, fodder and recently has been targeted for biomass-based biofuel production. Sorghum productivity in marginal lands under water-deficit and low nutrient soils could be potentially improved through a higher root microbiome diversity (Alsabri et al. 2020). Plant growth-promoting bacteria (PGPR) and arbuscular mycorrhizal fungi are known to colonize sorghum roots under water-deficit condition and improve plant growth and stress tolerance (Symanczik et al. 2018; Carlson et al. 2020). Sorghum roots colonized by arbuscular mycorrhizal fungi (AMF) were shown to have increased plant height, dry weight, and nitrogen and phosphorus contents (Nakmee et al. 2016). A recent study showed sorghum, under drought stress, specifically enriched Actinobacteria colonization in its rhizosphere (Xu et al. 2018). Parallel studies on artificial inoculation with free living *Azospirillum brasilense* enhanced the uptake of nitrate and potassium, resulting in improved grain yield in sorghum (Matiru and Dakora 2004).

Here, we used a visual screening method and identified an induced root secretion system mutant *Red root* (*RR*) by screening a collection of 256 EMS mutant lines. Genetic analysis showed that the *RR* locus is a dominant mutant with increased phenolic compound accumulation in roots. Further investigation on the role of *RR* root exudates in maintaining or altering the sorghum rhizosphere microbiome showed the enrichment of Actinobacteria under the well-watered condition. The current study also showed that the rhizosphere of BTx623 genotype was enriched with Actinobacteria under the water-deficit condition, which were reported earlier in sorghum (Xu et al. 2018), suggesting that the *RR* genotype might be subjected to stress naturally under the well-watered condition. Identification of the novel *RR* mutation with induced secretion system that alters the rhizosphere microbiome composition will offer an excellent opportunity to investigate the relationship between metabolites and microbial abundance.

## Materials and methods

### Generation of mutants and isolation of Red root (*RR*) mutant

BTx623, a public inbred line of sorghum (Miller 1977), was purified (self-fertilized) for six generations and used for chemical mutagenesis by ethyl methanesulfonate (EMS) (Xin et al. 2008). The BTx623 seeds were treated with EMS mutagen, washed with water, and planted in the field, at a density of about 120,000 seeds per hectare. The mutant lines were advanced using a single seed selection method to produce M_4_ population. A subset of the M_4_ genome sequenced population (250 lines), (Jiao et al. 2016), were germinated on non-sterile paper towels in the absence of light in growth chambers maintained at 28/25 °C as day/night temperatures. Non-sterile filter paper was used to create a microbial environment to visually observe any change in root color. From the germinated seedlings, a mutant line was identified with a unique red root phenotype. This mutant was named *RR* for its characteristic root coloration. The *RR* mutant was backcrossed with the parental BTx623 line to produce the BC_1_F_2_ population, then selfed to obtain the BC_1_F_2:3_. The homozygous dominant *RR* line was isolated from BC_1_F_2:3_ population. The homozygous dominant BC_1_F_2:3_ *RR* plants along with BTx623 plants were grown in the field for the rhizosphere microbiome sample collection and analysis.

### Experimental site, design, growth conditions and sample collection

Sorghum plants were grown in the Agriculture Experiment Station of the Agriculture Research Service of the United States Department of Agriculture (USDA-ARS) at Lubbock, Texas (33′39′N, 101°49′W) in May 2018. The field was composed of Amarillo fine sandy loam soil that was fertilized with bulk ammonium sulfate [(NH_4_)_2_SO_4_] and mono ammonium phosphate (NH_4_H_2_PO_4_) with an application level of 65 kg nitrogen and 27 kg phosphorus per hectare prior to planting. The seeds of BTx623 and *RR* mutant lines were planted in the plots of 4.67 m long with 1 m row spacing between each plant. A John Deere MaxEmerge planter pre-set with a seed density of approximately 17 seeds per m was employed for planting at a depth of approximately 3 cm. For well-watered condition plots, irrigation was supplied approximately 25 days after emergence with sub-surface irrigation drip at 3 mm per day, while the rain-fed plots were dependent only on rainwater and described from here onwards as water-deficit condition. Lubbock, Texas received a total of 381 mm of precipitation in 2018 and 123.698 mm of precipitation in the study period (June–October).

Rhizosphere samples of BTx623 and *RR* plants were collected at the flowering stage for soil microbial genomic DNA extraction (from three independent biological replicates). Sterile gloves were worn during the sample collection. First, the soil was dug around the plant to a depth of 0–10 cm with the help of a soil auger with care taken to avoid damage to the root and root hairs. Bulk soil attached to the roots was removed and the rhizosphere soil samples (defined as soil adhering to the root surface) of BTx623 and *RR* mutant lines were collected at different positions by gently cutting 10–15 root pieces. The root samples were collected in three biological replicates in 50 mL falcon tubes containing filter-sterilized epiphyte removal buffer [(0.75% monopotassium phosphate (KH_2_PO_4_) (Fisher BioReagents, Waltham, MA, USA), 0.95% dipotassium hydrogen phosphate (K_2_HPO_4_) (Fisher BioReagents), 1% Triton X-100 (Laboratory grade, Batavia, IL, USA) in double-distilled H_2_O)] (Xu et al. 2018). After collection, these samples were transported to lab on ice, which approximately took 2 h. The 50 mL tubes with samples were vortexed to remove the soil attached to the root tissue, the roots were discarded and the slurry was centrifuged at 6000*g* for 5 min at 4 °C to sediment the rhizosphere soil. The supernatant was discarded and the soil pellet representing rhizosphere sample was stored at − 80 °C. Sterile conditions and cold temperatures were maintained throughout the process of handling plant and soil material from the field to genomic DNA extraction for next-generation sequencing.

### Genomic DNA extraction and sequencing

Total genomic DNA was extracted from each rhizosphere sample and sequenced using bacterial (16S r-RNA, ribosomal RNA) and fungal (ITS2, Internal transcribed spacer) markers. The DNA was extracted from 0.25 g of rhizosphere soil samples using a power bead-based DNeasy^®^ Power Soil Kit^®^ (Qiagen, Valencia, CA, USA) following manufacturer’s instructions. The extracted genomic DNA samples were quantified by Nanodrop spectrophotometer at 260 nm and 1 µg of each sample was submitted for microbial analysis at the Microbial Analysis Resources and Services (MARS) facility of the University of Connecticut for sequencing (https://mars.uconn.edu/). The 16S r-RNA primers were designed with Illumina adapters and dual indices with 8 base pair Golay codes on 3′ and 5′ ends (Caporaso et al. 2012). The forward primer 515F and reverse primer 806R were used to amplify the V4 region of the 16S r-RNA. The fungal marker ITS2 and the primers ITS3 and ITS4 followed a similar design of V4 primers in having adapters and dual indices and were used to amplify ITS2 gene (White et al. 1990).

The rhizosphere genomic DNA samples were processed and sequenced at the MARS facility. Multiplexed sequences were separated using bcl2fastq program. The resultant sequences were further processed by the Mothur v.1.39.4 program (Kozich 2013). The sequences that did not meet minimum length requirements or with no exact matches were eliminated. For alignment of sequences, Silva nr_v119 alignment was used (Quast et al. 2012). RDP Bayesian classifier (Wang et al. 2007) was used against the Silva nr_v119 taxonomy database to sort the data by Operational Taxonomic Units (OTUs) providing the taxonomic identification of the microbes. The data were analyzed for comparing OTUs and compositional differences in bacterial and fungal communities of the rhizospheres of *RR* and BTx623 plants. Further, OTU data was used to perform alpha and beta diversity analysis using the Mothur program. The diversity values and distance matrices generated by Mothur were used in the R program to plot graphs with the inbuilt packages including betadisper, vegan, dplyr and ggplot2. Tukey HSD statistical analysis was performed with the alpha and beta diversity values to obtain *P* values for comparisons between *RR* and BTx623 genotypes under well-watered and water-deficit conditions. *P* < 0.1 was considered as significant for alpha–beta diversity analysis and *P* < 0.05 was considered as significant for abundance analysis.

### Estimation of root phenolics and anthocyanin content

Root tissue was collected from 10-day-old seedlings of BTx623 and *RR* mutant plants grown in the dark condition in growth chambers maintained at 28/25 °C as day/night temperatures. The harvested root tissues were frozen in liquid nitrogen and ground into a fine powder using mortar and pestle. Then the root powder was extracted using various organic solvents such as acidified methanol (99:1, v/v, of 100% methanol: HCl), 100% methanol, 100% ethanol and water to find the most efficient method. The extraction process was performed overnight using a shaker at 4 °C. Acidified methanol extraction resulted in the efficient extraction of the red colored metabolites from the root tissue and hence used for all extractions. Following the acidified methanol step, 100 µL of the extracted root sample was mixed with 100 µL of Folin-Ciocalteu reagent and incubated for 2 min at room temperature (Cicco et al. 2009). After incubation, 800 µL of 5% (w/v) sodium carbonate solution was added and the solution was thoroughly mixed and incubated at 40 °C for 20 min. Then, the mixtures were cooled on ice for 5 min and the resulting color was measured at 740 nm using a spectrophotometer. A standard curve was generated using serial dilutions of caffeic acid of 0.1, 0.01–0.001 mg/mL to calculate the unknown concentrations of the phenolic compounds. Total anthocyanin levels were measured using spectrophotometer (BioSpectrophotometer^®^ Kinetic, Eppendorf) absorbance readings at 529 nm.

### Root anthocyanin profile analysis using high-performance liquid chromatography (HPLC)

Agilent HPLC 1200 series with a standard Zorbax SB-C18 column (3.5 μl, 4.6*150 mm) was used for root anthocyanin profile analysis (Welch et al. 2008). The mobile phase was composed of 33% (A) (acetonitrile) and 67% (B) (milli-Q water-pH 2.1) and the flow rate was 1.5 mL/minute. Anthocyanin standards (apigenin, luteolin apigenidin and luteolinidin) that are commonly found in sorghum and available from ChromaDex (https://standards.chromadex.com/) were used in this study. These standards were diluted to 1 mg/mL concentration using the extraction solvent [(methanol acidified with 99:1, v/v, hydrochloric acid (HCl)] and analyzed using HPLC to obtain the retention times. The root extracts prepared from 10-day-old seedlings were analyzed under similar conditions using HPLC. The results of UV absorbance at different wavelengths (280 nm, 320 nm, 520 nm) were collected using spectrophotometer (Eppendorf). The retention peaks at 520 nm correspond to the anthocyanin derivatives. The HPLC analysis was repeated 3 times from 3 independent biological replicates for BTx623 and *RR* mutant root samples including 3 technical replicates for each biological replicate.

### Growing RR and WT genotypes on MS media with and without field soil water extracts

Half strength Murashige and Skoog (MS) media were prepared for 300 mL in 500 mL glass bottles, set pH to 5.7, added phytagel at 0.5% concentration, autoclaved at 121 °C for 30 min, about 25 mL of sterilized media was poured into glass tubes (20 cm long and 2.5 cm diameter) and solidified overnight in the laminar hood. To disinfest the seeds, 25 mL of 3.5% hypochlorite bleach was added to the seeds, incubated at room temperature for 30 min on a shaker, washed 3 times with water and dried on Petri dish with blotting papers in the laminar hood. BTx623 and *RR* sorghum seeds were further surface-disinfested by placing seeds in 10 mL of fungicide for 10 min and air-dried overnight in the laminar hood. For the sterile growth experiment, surface disinfested seeds were placed on the surface of the MS media in the glass tubes using sterile tweezers in the laminar hood. The glass tubes were then closed with the screw caps and transferred to a growth chamber that was maintained at 28/25 °C as day/night temperatures. Aseptic technique was used throughout the experimental procedure. For preparing field soil water extracts, 1 g of field soil was collected and placed in a 50 mL falcon tube with 50 mL sterile water and thoroughly mixed by placing on a rocker for 2 h at room temperature. Then, 50 μL of the soil extract was added to the sterile MS media in the glass tubes onto the seeds 3 days after the germination. Control set of seeds of BTx623 and *RR* genotypes were grown on sterile MS media without adding soil extract. The seedlings were allowed to grow on media for up to 8 days and images were taken. Fungal growth can be seen from day 5 (highlighted with pointed arrows in Fig. 3). These set of experiments were repeated three times.

### Statistical analyses

#### Principal component analysis (PCA)

Principal component analysis plots were drawn using ClustVis online R program. ClustVis is an online tool used for clustering the data with multiple variables and representing PCA plots using PCA loadings generated by input values of the relative abundance of major bacterial and fungal phyla (Metsalu and Vilo 2015). Principal component calculations in the ClustVis program were based on the R package PCA methods that uses the density model with inbuilt ggplot2 (8) packages, which were used for generating PCA plots. Unit variance and SVD imputation parameters were used to generate PCA plots.

#### Stacked bar graphs of bacterial and fungal relative abundances

The relative abundance percent values of major bacterial and fungal phyla were obtained from the krona files (Suppl. Fig. S4 and S5). Average relative abundance values of three biological replicates of each sample were used for all major bacterial and fungal phyla comparisons. The stacked bar graphs were generated in Microsoft Excel using 2D graphs. The difference between major bacterial and fungal phyla relative abundance were represented in separate graphs in both well-watered and water-deficit conditions.

#### Analysis of variance and student *t *test

Analysis of variance (ANOVA) with post hoc Tukey’s analysis was used to test the significant difference between alpha and beta diversity values calculated from relative abundances of *RR* and BTx623 rhizosphere samples. Student’s *t*-test (one tail and type 2) was used to calculate significantly enriched OTUs between BTx623 and *RR* plants* rhizospheres* under well-watered and water-deficit conditions.

#### Chi-square goodness of fit test

To determine the heritability pattern of the *RR* locus, segregation data from 200 dark-red colored *RR* seedlings were used to calculate the chi-square statistic values. The homozygous seedlings were identified using root color as a phenotype. The chi-square values were tested against the chi-square table for the probability value of *P* < 0.05, chi-square statistic value *x*^2^ = 3.841 with df  =  1.

## Results

### Isolation, phenotyping and genotyping of novel sorghum *RR* mutant

**Fig. 1:**
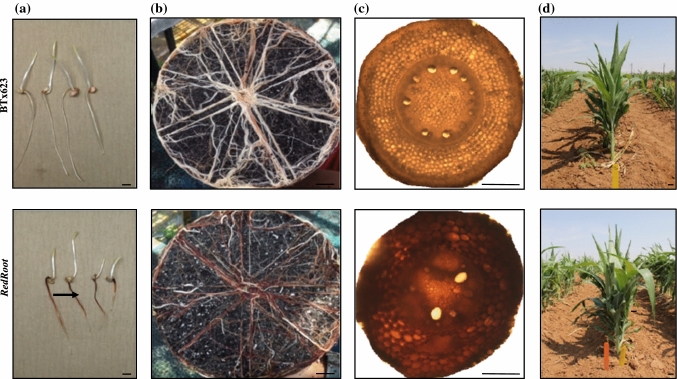
Identification of the novel *RR* mutant from sorghum EMS population. Upper panel shows the BTx623 phenotype and lower panel shows the *RR* phenotype. Roots accumulated red colored pigments. **a** Seedling stage. **b** Roots at the flowering stage. **c** Cross section of crown root showing the distribution of red pigments in all cell types of *RR* roots. **d** BC_1_F_2:3_ lines of BC1F2:3 lines of Red root and BTx623 were grown in the field for the rhizosphere microbiome analysis were grown in the field for the rhizosphere microbiome analysis. Scale bar represents 5 cm (**b, d**) and scale bar represents 0.3 cm (**a**, **c**)

Screening of mini-core sorghum lines (Jiao et al. 2016) by germination on non-sterile paper towels under dark condition resulted in the identification of *RR* mutant (Fig. 1a). The mutant accumulated red colored pigment in the root as well as secreted onto the paper towel (Suppl. Fig.S1a). Phenotyping of 2-months-old greenhouse-grown plants showed a uniform red colored root phenotype with a much darker appearance compared to BTx623, which showed a patchy red root phenotype (Fig. 1b). Additionally, the cross section of the crown root also confirmed a uniform distribution of the red pigment in all root cell types of the *RR* plants (Fig. 1c). To understand the inheritance pattern, the *RR* mutant was crossed with its parent BTx623 male sterile line (*ms8*), and the resultant BC_1_F_1_ plants showed the *RR* phenotype, whereas the BC_1_F_2_ plants showed a dominant segregation for *RR* mutation (Suppl. Fig. S1b). Furthermore, a possible dosage effect was observed in the root coloration of segregating *RR* plants with dark-red, medium-red and pale-red roots (Suppl. Fig. S2). Dark-red roots showed reduced root length (3 cm) in comparison to the wild-type (5.6 cm) and medium-red roots (5.5 cm) or pale-red roots (4.75 cm) (Suppl. Fig. S2). The BC_1_F_2_ *RR* plants with red colored roots were further advanced to BC_1_F_2:3_ by selfing to obtain the homozygous dominant plants from the heterozygous dominant plants. The homozygous BC_1_F_2:3_ seeds were planted in the field for studying the effect of *RR* genotype on the rhizosphere microbiome composition (Fig. 1d).

### *RR* mutant roots accumulated higher levels of pigmented compounds

**Fig. 2:**
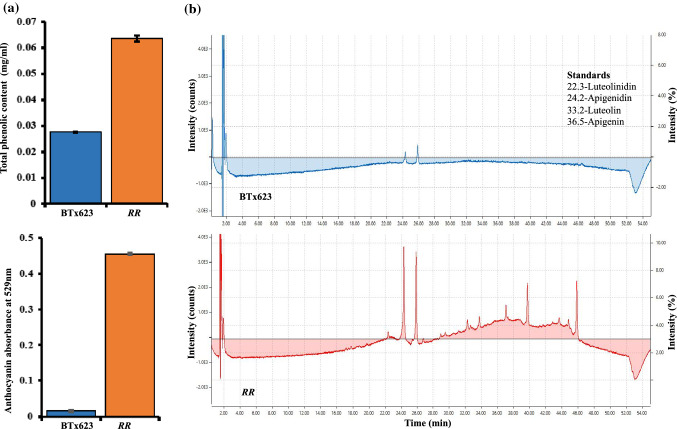
Metabolic profiling of total root extracts of BTx623 and *RR* mutant. **a** Total phenolic content of the root extracts of BTx623 and *RR* seedlings. Total anthocyanin content of root extracts was measured at 529 nm using spectrophotometer. **b** HPLC analysis of acidified methanol root extracts of BTx623 and *RR* genotypes showed retention times of peaks that are different from known anthocyanin standards (luteolinidin, apigenidin, luteolin and apigenin). The retention time of known anthocyanin derivatives used in this study was listed as subset text within the figure

Red root plants showed a red root phenotype; however, the nature of the compounds that were causing the *RR* phenotype was not known. To identify the secreted root pigments (anthocyanins or any other secondary metabolites), phenolic assay and chromatography methods were employed. The results of Folin-Ciocalteu reagent assay (Singleton et al. 1999) showed a ten-fold increase in phenolic compounds in *RR* roots than in BTx623 roots (Fig. 2a). The absorbance of anthocyanin pigments at 529 nm also displayed a greater accumulation of anthocyanin related compounds in *RR* plant roots relative to BTx623 roots (Fig. 2a). To understand the composition of these anthocyanin related compounds, the methanol extracts of total root from both BTx623 and *RR* were analyzed using HPLC. The resultant retention peaks at 520 nm (anthocyanin absorbance range) were compared with the retention peaks of the known sorghum associated anthocyanin derivative standards (apigenin, apigenidin, luteolin and luteolinidin) (Mueller‐Harvey and Reed 1992; Mizuno et al. 2016). The results of HPLC of *RR* root extracts indicated the presence of peaks with different retention times compared to the peaks of known anthocyanin derivative standards (Fig. 2b). These data suggest that the anthocyanin derivatives in *RR* roots might be glycosylated or methylated forms of known anthocyanin derivatives.

### *RR* phenotype is only induced under non-sterile conditions

**Fig. 3:**
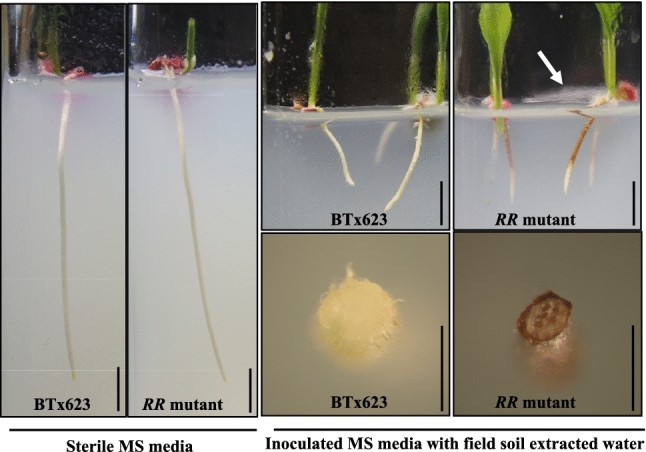
Induced secretion system of the *RR* mutant. Surface disinfested BTx623 and *RR* seeds were grown on sterile MS media (left panel) with and without field soil water extracts (right-top panel) in the growth chambers maintained at 28/25 °C as day/night temperatures. The root images were taken on the 8th day of post germination. Cross section of the root collected from MS media grown seedlings with field soil water extract showed dark red root phenotype only in the *RR* genotype (right-bottom panel). Scale bar represents 1 cm

The soil-grown *RR* plants showed a red root phenotype; however, it was not known if it is a constitutive or an induced phenotype. This was tested by germinating BTx623 and *RR* seeds on sterile MS media with and without soil extracts of field soil (Fig. 3). The *RR* mutants and BTx623 grown on sterile media had similar root lengths, whereas *RR* roots inoculated with soil water extract showed reduced root length in comparison to inoculated BTx623 roots (numerical data not shown). The *RR* plants grown on sterile medium did not show any difference in the root color phenotype but developed red color exclusively in MS medium inoculated with the field soil water extract (Fig. 3). Fungal growth was observed only in MS media glass tubes with field soil inoculant suggesting the role of soil factor (microbes or microbial secretions or other factors) in the induction of red root secretion (white arrow pointed in Fig. 3).

### Diversity analysis

#### Bacterial diversity is altered in *RR* mutant rhizosphere

Root exudates play an important role in the composition of the rhizosphere microbiome (Shiaris et al. 2009; Mönchgesang et al. 2016). Since the *RR* mutant showed higher phenolic compounds in the soil-grown plant roots, the role of these phenolic compounds in determining the rhizosphere microbiome composition was investigated by metagenomics analysis.

#### *RR* rhizosphere maintained higher overall bacterial species number and diversity than BTx623

##### Well-watered condition

**Fig. 4:**
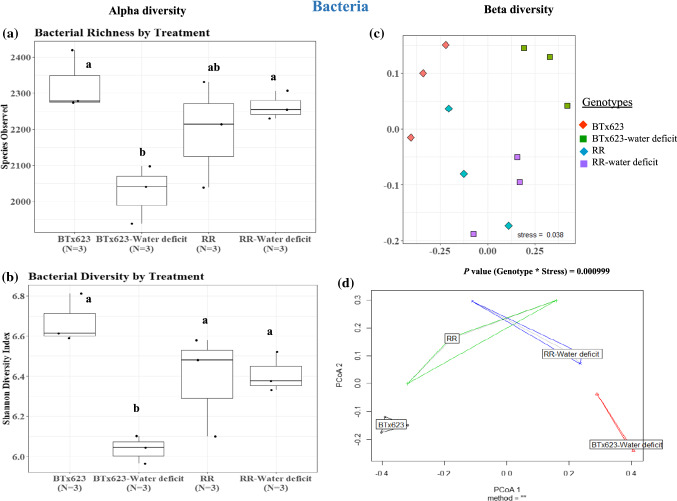
Bacterial alpha and beta diversity analysis. Alpha diversity analysis of species richness and Shannon diversity index of rhizosphere samples of both genotypes under well-watered (BTx623 and *RR*) and water-deficit (BTx623-water-deficit and *RR-*water-deficit) conditions showed a significant change in bacterial species number and diversity **(a**, **b)** (*P* ≤ 0.1). The beta diversity analysis (theta dissimilarity index) and beta dispersity analysis also showed a significant change in overall bacterial species abundance (**c**, **d**) between BTx623 and *RR* rhizosphere samples (*P* ≤ 0.1). *P* values were calculated using analysis of variance with post-hoc Tukey’s test

The alpha and beta diversity analysis of bacterial species from the rhizosphere of *RR* and BTx623 lines (Fig. 4 and Suppl. Table S1) indicated that there was no significant change in the total number of bacterial species in the well-watered condition (*P* = 0.32, Fig. 4a, Suppl. Table S1). The Shannon diversity index, which indicates species diversity within each genotype, also did not show any significant difference between BTx623 and *RR* rhizospheres under the well-watered condition (*P* = 0.18, Fig. 4a, Suppl. Table S1). This demonstrated that the overall number of bacterial species and species diversity did not alter between BTx623 and *RR* rhizospheres under the well-watered condition.

##### Water-deficit condition

The bacterial species between BTx623 and *RR* rhizospheres in water-deficit condition varied in the total number of bacterial species (Fig. 4a, Suppl. Table S1). *RR* rhizosphere under the water-deficit condition had a higher number of bacterial species than the BTx623 rhizosphere under the water-deficit condition (*RR*-water-deficit vs BTx623-water-deficit, *P* = 0.062, Fig. 4a). A similar trend was observed with the Shannon diversity index, which showed a higher bacterial diversity in the rhizosphere of *RR* genotype than in BTx623 rhizosphere under the water-deficit condition (Fig. 4b, *RR*-water-deficit and BTx623-water-deficit, *P* = 0.068). These data suggest that the water-deficit condition has a negative effect on bacterial species number and diversity in the BTx623 rhizosphere, but not in the *RR*-rhizosphere.

A similar pattern was also observed within each genotype between well-watered and water-deficit conditions. Under the water-deficit-condition, there was a significant reduction of the overall bacterial diversity within the genotype of BTx623 (Fig. 4b, BTx623-water-deficit vs BTx623, *P* = 0.021), whereas no significant change was observed for the *RR* genotype (Fig. 4b, *RR*-water-deficit vs *RR*, *P* = 0.80). This shows that *RR* genotype maintains a higher bacterial diversity than the BTx623 rhizosphere under the water-deficit condition.

### Beta diversity analysis using Jaccard distance and Theta-YC tests

Further analysis of Beta diversity for dissimilarity among samples using Jaccard distance and Theta-YC tests showed that the bacterial abundance varied significantly (Genotype * Stress *P* = 0.0009) between these two genotypes in both well-watered and water-deficit conditions (Fig. 4c, Suppl. Table S1). Further, Tukey’s Post-hoc analysis on distance matrices also indicated statistical difference (*P* = 0.1) in bacterial species diversity between BTx623 and *RR* in both well-watered and water-deficit conditions (Suppl. Table S1). Overall, the beta-dispersity plot showed that the bacterial composition was more heterogeneous in the rhizosphere of *RR* genotype, both under well-watered and water deficit conditions, whereas in case of BTx623 rhizosphere, the water deficit created more heterogenous composition than the well-watered BTx623 (Fig. 4d). This indicated that even under well-watered condition, *RR* rhizosphere was associated with more heterogeneous bacterial composition in comparison with well-watered BTx623, and this can be attributed to the root exudate phenotype exclusively present in the *RR* mutant plants.

### Fungal diversity did not vary between *RR* and BTx623 rhizospheres under both water regimes

**Fig. 5:**
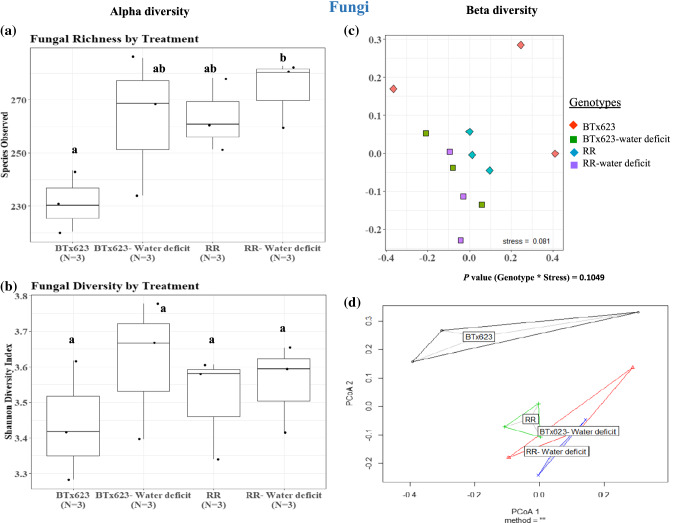
Fungal alpha and beta diversity analysis. Alpha diversity analysis of species richness and Shannon diversity index of rhizosphere samples of both genotypes under well-watered (BTx623 and *RR*) and water-deficit (BTx623-water-deficit and *RR-*water-deficit) conditions showed no significant difference in fungal species number and diversity **(a**, **b).** The beta diversity analysis (theta dissimilarity index) and beta dispersity analysis showed no significant difference in overall fungal species abundance (**c**, **d**) between the genotypes and water regimes. *P* values were calculated using analysis of variance with post-hoc Tukey’s test

Analysis of total fungal species in the rhizosphere demonstrated that the fungal diversity did not change significantly between BTx623 and *RR* rhizospheres under the well-watered condition (*RR* vs BTx623, *P* = 0.18, Fig. 5a, Suppl. Table S1) nor in the water-deficit condition (*RR*-water-deficit vs BTx623-water-deficit, *P* = 0.85, Fig. 5a). The Shannon diversity index showed no significant difference in fungal diversity in either BTx623 or *RR* plants grown under water-deficit condition (Fig. 5b), indicating that water availability has no effect on fungal species diversity between these genotypes. Beta diversity analysis showed that there was no significant difference (*P* = 0.10) in fungal species abundance between the genotypes under both water conditions (Fig. 5c, Suppl. Table S1). A similar pattern was observed with the beta-dispersity analysis, in which except BTx623, all other conditions are overlapped (Fig. 5d, Suppl. Table S1). Overall, data suggest that the rhizospheres of *RR* plants were not altered in fungal diversity in comparison to BTx623 under well-watered or water-deficit conditions.

### Relative abundance analysis

**Fig. 6:**
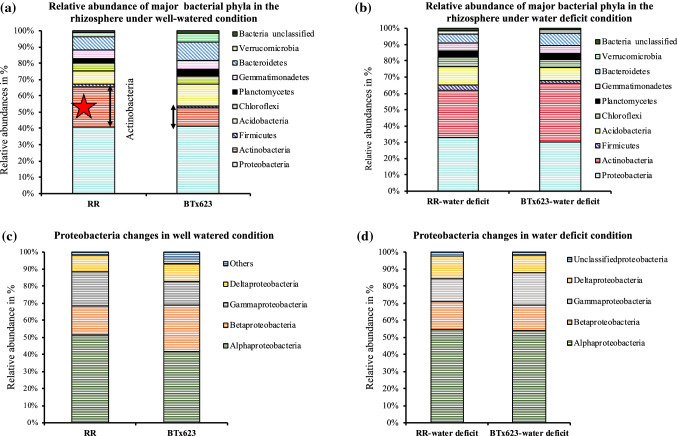
Relative abundances of bacterial phyla and classes in *RR* and BTx623 rhizospheres. Major bacterial communities (Proteobacteria, Actinobacteria, Firmicutes, Acidobacteria, Chloroflexi, Planctomycetes, Gemmatimonadetes, Bacteroidetes, Verrucomicrobia and Bacteria unclassified) were compared between *RR* and BTx623 rhizospheres under well-watered and water-deficit conditions. **a** *RR* rhizosphere showed higher levels of Actinobacteria compared to BTx623 rhizosphere under the well-watered condition. **b** Comparison of relative abundance values of bacterial phyla in *RR* and BTx623 under the water-deficit condition. **c** Comparison of relative abundance values of classes of Proteobacteria under the well-watered condition. **d** Comparison of relative abundance values of classes of Proteobacteria (Alpha, Beta, Gamma and Deltaproteobacteria) under the water-deficit condition. The data used here for comparison is the mean of the relative abundance values of all the three biological replicates in both *RR* and BTx623 rhizosphere samples. These relative abundance values of major bacterial phyla between both genotypes were represented in the form of stacked bars using Excel. *RR* and BTx623 labels represent the rhizosphere samples under the well-watered condition, while *RR*-water-deficit and BTx623-water-deficit labels represent the rhizosphere samples under the water-deficit condition

**Fig. 7:**
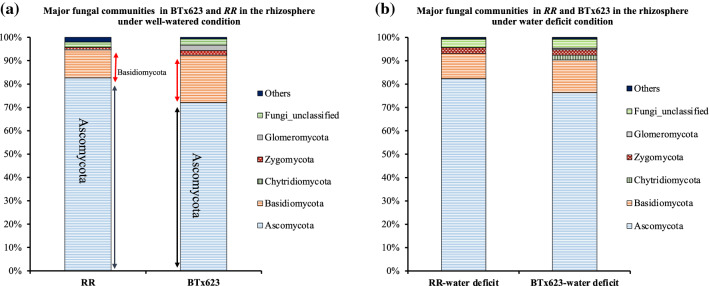
Relative abundances of fungal phyla in *RR* and BTx623 rhizospheres. Comparison of relative abundance values of major fungal communities (Ascomycota, Basidiomycota, Chytridiomycetes, Zygomycota, Glomeromycota, fungi unclassified and others) between rhizospheres of *RR* and BTx623 genotypes under well-watered and water-deficit conditions. **a** Comparison of major fungal phyla relative abundance values between *RR* and BTx623 under the well-watered condition. **b** Comparison of major fungal phyla relative abundance values between *RR* and BTx623 under the water-deficit condition. Relative abundance values of fungal phyla were collected from krona files, and the mean of the relative abundance values of three replicates of both genotypes was used to compare rhizosphere samples of both genotypes. *RR* and BTx623 labels belong to the rhizosphere samples under the well-watered condition, whereas *RR*-water-deficit and BTx623-water-deficit labels belong to the rhizosphere samples under the water-deficit condition

**Fig. 8:**
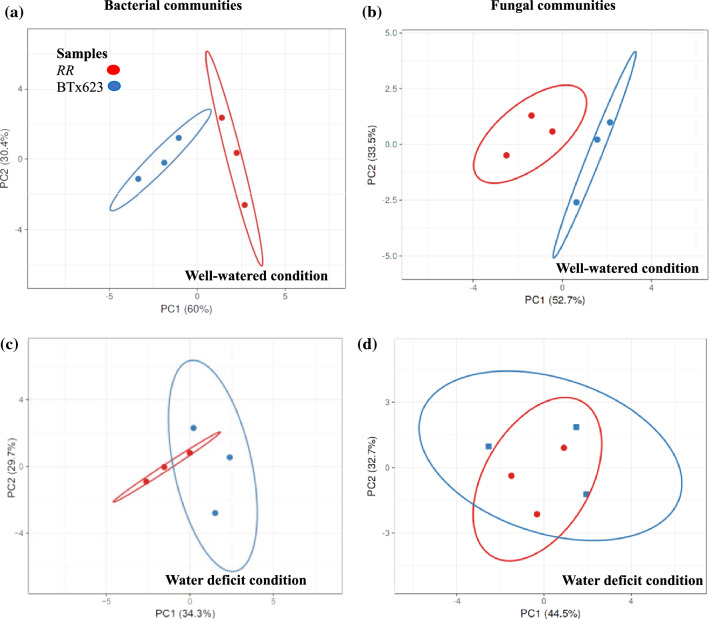
Principal Component Analysis (PCA) of bacterial and fungal phyla relative abundance values. PCA of the relative abundance values of major bacterial communities and major fungal communities showed differences in *RR* and BTx623 rhizospheres under well-watered condition and water-deficit conditions. **a** Comparison of major bacterial communities between the rhizosphere samples of *RR* and BTx623 genotypes under the well-watered condition showed separate clusters indicating variation. **b** Comparison of major fungal communities between BTx623 and *RR* rhizospheres under the well-watered condition also showed variation with separate clusters. **c** Comparison of major bacterial communities between the rhizosphere samples of *RR* and BTx623 genotypes under the water-deficit condition showed overlapped clusters indicating less variation. **d** Comparison of major fungal communities between BTx623 and *RR* rhizospheres under the water-deficit condition showed overlapped clusters representing less variation

The relative abundance of specific microbial communities was investigated between *RR* and BTx623 plant rhizospheres, then the differences in predominant bacterial and fungal phyla under both water regimes were analyzed (Figs. 6, 7, 8, and Suppl. Table S2).

### Principal component analysis of major bacterial and fungal phyla of both genotypes vary in the well-watered condition but overlaps in the water-deficit condition

Principal component analysis was used to analyze the total variation in the relative abundances of major bacterial and fungal phyla present in the rhizospheres of *RR* and BTx623 genotypes. The PCA analysis of the mean relative abundance values of nine major bacterial phyla and unclassified phyla showed two separate clusters with PC1 (60%) and PC2 (30.4%) under the well-watered condition (Fig. 8a), whereas they overlapped under the water-deficit condition with PC1 (34.3%) and PC2 (29.7%) (Fig. 8c). The PCA analysis of major fungal phyla in both genotypes also showed two separate clusters with PC1 (52.7%) and PC2 (33.5%) under the well-watered condition (Fig. 8b), whereas they overlapped under the water-deficit condition PC1 (44.5%) and PC2 (32.7%) (Fig. 8d). Separate clusters indicated distinct differences between BTx623 and *RR* genotypes under the well-watered condition. Overlapped clusters under the water-deficit condition indicated reduced differences in the relative bacterial and fungal abundances between BTx623 and *RR* genotypes. This data showed that water-deficit condition reduces the differences in the relative abundances of both bacterial and fungal phyla in the rhizospheres of BTx623 and *RR* genotypes.

### Relative abundance comparison of major bacterial phyla in BTx623 and *RR* plant rhizospheres

#### Well-watered *RR* rhizosphere showed a higher relative abundance of specific classes of Actinobacteria and Alphaproteobacteria

Under well-watered condition, Actinobacteria abundance increased in the *RR* rhizosphere compared to the BTx623 rhizosphere (Fig. 6a, *RR* vs BTx623; 24.6% vs 11.3%, *P* = 0.10). A further analysis of the specific classes within Actinobacteria Phyla (Streptomycetales, Micrococcales, Frankiales and Micromonosporales etc.,) showed significant differences in Actinobacteria relative abundances in the rhizosphere of *RR* and BTx623 plants (Suppl. Table S2, *RR* vs BTx623; 82% vs 78%, *P* = 0.02). This indicates that the *RR* plant rhizosphere was enriched for Actinobacteria phyla microbes under the well-watered condition. Another major bacterial phylum, Proteobacteria, did not show any difference in relative abundance between BTx623 and *RR* plant rhizospheres under the well-watered condition (Fig. 6a, Suppl. Table S2). Further analysis of the bacterial classes within the Proteobacteria phyla showed a significant increase in Alphaproteobacteria (Fig. 6c, *RR* vs BTx623; 51.3% vs 41.6%, *P* = 0.03) and decrease in Beta proteobacteria abundance in *RR* rhizosphere compared to BTx623 rhizosphere (Fig. 6c, *RR* vs BTx623; 17% vs 27.3%, *P* = 0.002). This reflects that compensatory changes in relative abundance levels of different classes within the major phylum Proteobacteria could have resulted in no change in the overall abundance of specific phyla between BTx623 and *RR* rhizosphere samples. On the other hand, the rhizosphere of BTx623 plants displayed a higher abundance of Acidobacteria (*RR* vs BTx623; 8% vs 13%, *P* = 0.04), Planctomycetes (*RR* vs BTx623; 3% vs 4.3%, *P* = 0.04), and Verrucomicrobia (*RR* vs BTx623; 2.67% vs 5%, *P* = 0.004) in comparison to the *RR* rhizosphere.

#### Water-deficit condition enriched Actinobacteria, Alphaproteobacteria and Firmicutes in rhizospheres of *RR* and BTx623 genotypes

Analysis of the relative abundance showed that the water-deficit condition enriched with Actinobacteria in the rhizospheres of *RR* plants and BTx623 plants (Fig. 6b, Suppl. Table S2; *RR-*water-deficit: 29% vs *RR*: 25%; BTx623-water-deficit: 36% vs BTx623: 11%). Although the increase in the *RR* genotype in Actinobacteria was modest, the difference was distinct in the BTx623 genotype under the water-deficit condition (Suppl. Table S2). The water-deficit condition decreased the total Proteobacteria abundance in the rhizospheres of both genotypes (*RR*-water-deficit: 32% vs *RR*: 36%; BTx623-water-deficit: 30% vs BTx623: 40%). Further analysis of classes of Proteobacteria showed differences in the sub classes of Alpha, Beta, Gamma and Deltaproteobacteria. Alphaproteobacteria were enriched in the rhizospheres of *RR*- and BTx623 plants under the water-deficit condition (*RR*-water-deficit: 55% vs *RR*: 51%; BTx623-water-deficit: 54% vs BTx623: 42%) (Fig. 6d, Suppl. Table S2), while Betaproteobacteria levels were reduced only in the rhizosphere of BTx623 genotype (BTx623-water-deficit: 15% vs BTx623: 27%; *RR*-water-deficit: 16% vs *RR*: 17%). The Deltaproteobacteria abundance in the rhizosphere of BTx623 under the water-deficit condition remain unchanged (BTx623-water-deficit: 10.33% vs BTx623:10%) and the Gamma proteobacteria showed enrichment to 19% (BTx623:13%). The trend was different in the rhizospheres of the *RR* genotype where Deltaproteobacteria increased (*RR*-water-deficit: 13% vs *RR*: 10%) and Gammaproteobacteria decreased (*RR*-water-deficit: 13% vs *RR*: 20%). Acidobacteria levels were increased in the *RR* rhizosphere and decreased in the BTx623 rhizosphere under the water-deficit condition (*RR*-water-deficit: 11% vs *RR*: 8%; BTx623-water-deficit: 8% vs BTx623: 13%). The *RR* mutant also showed a higher relative abundance of Firmicutes under the water-deficit condition (*RR*-water-deficit vs BTx623-water-deficit; 3% vs 1%, *P* = 0.067286) (Fig. 6b and Suppl. Table S2).

#### Effect of water-deficit condition on rhizosphere bacterial abundance within the genotypes

Rhizosphere of BTx623 plants under water-deficit condition showed a significant increase in Actinobacteria abundance in comparison to the BTx623 plants rhizosphere grown under well-watered condition (BTx623-water-deficit: 35.6% vs BTx623: 11.3%, *P* = 0.005). In addition, the BTx623 rhizosphere under the well-watered condition showed higher abundance of three major bacterial phyla including Acidobacteria, Planctomycetes, and Verrucomicrobia, that were reduced under the water-deficit condition (BTx623-water-deficit vs BTx623, Suppl. Table S2). A similar reduction trend was not observed in *RR* rhizosphere in both water regimes (*RR*-water-deficit vs *RR*, Suppl. Table S2). Moreover, Proteobacteria classes significantly increased in Alphaproteobacteria and the abundance of Betaproteobacteria in rhizospheres of BTx623 under the well-watered condition was reduced when compared to the rhizosphere of BTx623 under the water-deficit condition (BTx623 vs BTx623-water-deficit). However, this pattern was not observed in the rhizosphere of *RR* genotype under both water conditions (*RR* vs *RR*-water-deficit, Suppl. Table S2). This explains that the effect of water-deficit condition on the abundance of these bacteria was more evident in the BTx623 rhizosphere than in the *RR* rhizosphere.

#### Relative abundance differences in major fungal Phyla in rhizospheres of the *RR* and BTx623 rhizospheres

To study the variation of major fungal phyla in the rhizospheres of *RR* and BTx623, the relative abundance of major fungal phyla was calculated for Ascomycota, Basidiomycota, Chytridiomycota, Zygomycota, Glomeromycota, unclassified fungi and others (Fig. 7b). Analysis of variance with Tukey’s post hoc analysis showed no significant differences in fungal phyla between BTx623 and *RR* rhizospheres under both water conditions (Suppl. Table S2). Further, the major classes within the Ascomycota and Basidiomycota Phyla also did not differ in abundance between BTx623 and *RR* rhizospheres under both water conditions (Suppl. Table S2). This result is in accordance with no change in Shannon diversity index value and fungal beta diversity analysis between BTx623 and *RR* rhizosphere (Fig. 5f, g).

#### Most abundant bacterial and fungal OTUs (Operational Taxonomical Units) differ between *RR* mutant and BTx623

Under the well-watered condition, the total number of bacterial OTUs in the rhizosphere of BTx623 were 9437, while *RR* mutant had 8314 OTUs. The rhizosphere of BTx623 plants under the water-deficit condition showed 6281 bacterial OTUs, whereas *RR* rhizosphere under the water-deficit condition showed 7608 OTUs. The total fungal OTUs in both genotypes in well-watered condition (BTx623: 668 and *RR*: 748) and in water-deficit condition (BTx623-water-deficit: 717 and *RR*-water-deficit: 752) did not show significant differences (Suppl. Table S3).

#### Most abundant bacterial OTUs in rhizospheres of BTx623 and *RR* mutant

To further, understand the specific microbial interactions in the rhizosphere, the 50 most abundant OTUs in BTx623 and *RR* genotypes under well-watered and water-deficit conditions were analyzed.

##### Under well-watered condition

The 50 most abundant OTUs in rhizospheres of *RR* mutant plants are Actinobacteria (12), Proteobacteria (27), Acidobacteria (3), and Bacteroidetes (4) followed by other phyla. The 10 most abundant OTUs in the rhizosphere of the *RR* mutant showed OTU numbers 5, 6, 9, 10, 13, 18, 19, 22, 26 and 30 (Suppl. Table S3) of phyla Actinobacteria, Proteobacteria (alpha, beta and gamma), Acidobacteria and Bacteroidetes. In rhizosphere of BTx623, the 50 most abundant OTUs are in the phyla Actinobacteria (5), Proteobacteria (24), Acidobacteria (10) and Bacteroidetes (6). The 10 most abundant OTUs showed OTUs 5, 6, 9, 10, 15, 18, 19, 23, 24 and 28 that fall in the major bacterial phyla of Verrucomicrobia, Proteobacteria (Alpha, gamma and unclassified), Actinobacteria and Acidobacteria (Suppl. Table S3). This showed that the total OTU numbers of Actinobacteria are higher in the *RR* rhizosphere compared to the BTx623 rhizosphere (*RR*: 12; BTX623: 5). Applying the student *t*-test also identified 334 significantly different OTUs (at 95% confidence and significance below 0.005) between the rhizospheres of BTx623 and the *RR* mutant (Suppl. Table S3).

##### Under water-deficit condition

The 50 most abundant OTUs in the *RR* plants rhizosphere under water-deficit condition are Actinobacteria (14), Proteobacteria (21), Acidobacteria (4), Gemmatimonadetes (4) etc., among which the 10 most abundant OTUs showed OTUs 5, 6, 7, 9, 10, 13, 14, 15, 22 and 29 (Suppl. Table S3). The 50 most abundant OTUs in the rhizosphere of BTx623 plants under the water-deficit condition include those in Actinobacteria (12), Proteobacteria (27), Acidobacteria (3), and Bacteroidetes (4) among which the 10 most abundant OTUs are 5, 6, 7, 9, 10, 13, 14, 15, 20 and 21 (Suppl. Table S3). Further statistical analysis of total identified OTUs by student t-test identified 266 significantly different OTUs between BTx623 and *RR* plant rhizospheres under the water-deficit condition (Suppl. Table S3).

#### Most abundant fungal OTUs in the rhizospheres of *RR* mutant and BTx623

The predominant fungal OTUs in the rhizosphere of the *RR* and BTx623 plant rhizospheres under both well-watered and water-deficit conditions were analyzed.

##### Well-watered condition

The 50 most abundant fungal OTUs in the rhizospheres of the *RR* mutant included the phyla of Ascomycota (35), Basidiomycota (7), Glomeromycota (2) with the 10 most abundant OTUs belonging to 1, 2, 3, 4, 5, 6, 9, 11, 15 and 17 (Suppl. Table S3). In the rhizosphere of the BTx623 plant, the 50 most abundant OTUs included the phyla Ascomycota (36), Basidiomycota (10), Zygomycota (1) with the 10 most abundant OTUs of 1, 2, 3,6,7,8,10,11,13 and 15 (Suppl. Table S3). This showed that under the well-watered condition, there was no major difference in the total number of OTUs between the *RR* and the BTx623 plant rhizospheres. The student t-test showed significant changes only in 43 OTUs between BTx623 and *RR* rhizosphere suggesting that fungal OTUs did not vary in significant numbers with the change in the genotype (Suppl. Table S3).

##### Water-deficit condition

The 50 most abundant fungal OTUs in the rhizospheres of the *RR* plants under the water-deficit conditions were in the phyla Ascomycota, Basidiomycota, Zygomycota etc., with the 10 most abundant OTUs of 1, 2, 3, 4, 5, 8, 12, 19, 20 and 32 (Suppl. Table S3). Whereas, in the rhizospheres of BTx623 plants grown under the water-deficit condition, the 50 most abundant OTUs are in the phyla Ascomycota (38), Basidiomycota (7), Zygomycota (3) etc., among which the 10 most abundant OTUs in BTx623 showed OTUs of 1, 2, 3, 4, 5, 8, 10, 12, 14 and 16 (Suppl. Table S3). Student t-test showed that only 38 OTUs were significantly different between these two genotypes under the water-deficit condition (Suppl. Table S3).

## Discussion

Microbiome of plants acts as secondary genome as plants interact with a plethora of microbes in their lifecycle that colonize or inhabit in different compartments of roots such as rhizosphere, rhizoplane, endosphere, and phyllosphere which ultimately affects the plant growth, productivity, carbon sequestration and phytoremediation (Bulgarelli et al. 2012; Lundberg et al. 2012; Turner et al. 2013). Both endo and ecto-phytic microbiome associations in the root and rhizosphere play a prominent role in determining plant health, disease resistance, root pathogen suppression and in triggering endophytic colonization (Lugtenberg and Kamilova 2009; Berendsen et al. 2012). The rhizosphere microbiome is greatly influenced by the nature of the root exudates, mucilage and sloughed cells (Moe 2013). Root exudates are complex carbon compounds composed of low molecular weight primary metabolites such as sugars, amino acids, organic acids, and secondary metabolites such as phenols, flavonoids and terpenoids (Faure et al. 2009) which can alter soil properties and shape the rhizosphere microbiome (Yoneyama et al. 2008; Vives-Peris et al. 2020). Phenolic compounds such as flavonoids, and sugars such as arabinogalactans, are some of the known rhizosphere root exudates (Nguema-Ona et al. 2013; Koroney et al. 2016). Studies reported that phenolic compounds act as signaling molecules and associate with a higher number of unique OTUs than sugars and alcohol compounds in the root exudates (Badri et al. 2013). Legumes release flavonoids to signal nitrogen-fixing bacteria (Broughton et al. 2003) and some plant species release strigolactones, a sesquiterpene, to recruit symbiotic arbuscular mycorrhizal fungi in their microbiome (Yoneyama et al. 2008). Overall data from different plant species suggests that phenolic compounds play a significant role in shaping the rhizosphere microbiome composition.

The present study identified a sorghum mutant (*RR*) that produces higher amounts of pigmented compounds from roots compared to BTx623 genotype, especially in response to the presence of external stimuli in the soil. Further, *RR* showed altered bacterial composition in their rhizosphere compared to BTx623, which is consistent with the fact that intraspecific genetic variation is known to alter the rhizosphere microbiome composition (Lugtenberg and Kamilova 2009). Previous studies have shown that sorghum accumulates anthocyanin-like pigments in response to pathogen infection, known as “purple wound response” (Funnel and Pedersen 2006). In sorghum, wounding enhanced anthocyanin content in the mesocotyls and inoculation of *Fusarium* in non-wounded sorghum plants also induced anthocyanins, soluble phenolics and increased activities of peroxidases, chitinases, and β 1, 4 glucanases in the roots (Huang and Backhouse 2005). Further, an EMS sorghum mutant, RG (RED for GREEN) showed an excessive accumulation of anthocyanin derivatives in aboveground plant material (Petti et al. 2013). The combination of root exudate composition and microbial substrate uptake potential defines the rhizosphere microbiome diversity in an annual grass species (Zhalnina et al. 2018). Several recent studies have shown that there is a specific association between the phenolic secondary metabolite and abundance of microbial species. It was shown that benzoxazinoids in maize root exudate specifically enriched *Pseudomonas putida* (Neal et al. 2012) and oxylipins in tomato root exudate specifically enriched *Trichoderma harzianum* (Lombardi et al. 2018).

Similarly, environmental factors like drought, heat, soil composition and crop rotations are also shown to play a significant role in microbiome composition (Cavicchioli et al. 2019). The environmental conditions, such as drought stress alters plant root exudate composition in *Quercus ilex* (evergreen oak), thereby affecting the microbial interactions in the rhizosphere (Gargallo-Garriga et al. 2018). Drought enriches roots of many plant species with monoderm bacteria in the roots, hence postulated that the drought effects the overall plant metabolism which could be a factor in shaping microbiome diversity (Xu and Coleman-Derr 2019). Previously, it was reported that the effect of the water-deficit condition was associated with 15% reduction in the rhizosphere microbiome diversity in BTx642 and RTx430 (Xu et al. 2018), indicating the negative effect of the water-deficit condition on the rhizosphere microbiome composition. Interestingly, under the water-deficit condition, the rhizosphere of *RR* genotype retained a higher bacterial diversity than BTx623 rhizosphere, suggesting a potential role of altered root exudates in the *RR* mutant. It is possible that *RR* mutant shows an activated stress response under the well-watered condition and secretes the pigmented compounds. The present study analyzed only phenolics, it is possible that *RR* roots may secrete other carbohydrates or amino acids in its root exudates that could help to recruit or retain microbiome abundance under the water-deficit condition. Comprehensive biochemical composition analysis of the *RR* root exudate under the well-watered condition will reveal the reason behind the specific enrichment of bacterial species compared to BTx623. The higher abundance of Actinobacteria in the rhizosphere in *RR* plants suggests that these plants might naturally experience some level of stress under the well-watered condition. It is possible that *RR* mutant is relatively more drought-tolerant compared to BTx623 as the bacterial species that help withstand water-deficit condition, for example, monoderm bacteria, were more abundant in *RR* mutant rhizosphere (Fig. 6 and Suppl. Table S2).

In addition to the microbiome abundance, its composition plays a significant role in plant health and productivity. *RR* mutant had a higher abundance of Actinobacteria in its rhizosphere under the well-watered condition and their abundance was found to increase in the water-deficit condition, not only in *RR* but also in BTx623. This is consistent with the recent study that showed, under the drought condition, sorghum was found to enrich Actinobacteria community in its rhizosphere (Xu et al. 2018). Actinobacteria, is known to promote soil health by decomposing high molecular weight compounds to hydrocarbons, aids in nutrient cycling, triggers the production of metabolites, and promotes plant growth regulators. Actinobacteria also take part in the breaking down of plant cellulose by working in conjunction with other microorganisms (Bhatti et al. 2017). In addition to their roles in biogeochemical cycling, they are also involved in antibiotic production, nitrogen cycling (Zhang et al. 2019), improved seedling vigor, drought tolerance, and yield in monocot species (Yandigeri et al. 2012; Selim et al. 2019). Functions of Actinobacteria suggests that *RR* mutant may have additional advantages in growth and development compared to BTx623.

Apart from Actinobacteria, *RR* mutant also had enrichment of Alphaproteobacteria in the well-watered condition which was shown to enrich the rhizosphere of drought treated rice (Santos-Medellín et al. 2017) and pepper plants (*Capsicum annuum* L.) (Marasco et al. 2012). In general, Proteobacteria phyla comprise several PGPR (plant growth-promoting rhizobacteria) species that promote plant growth (Bruto et al. 2014). *RR* mutant also showed a higher relative abundance of Firmicutes under the water-deficit condition. Firmicutes belong to monoderm bacteria, similar to Actinobacteria, and are found to be generally enriched during the drought stress condition (Naylor and Coleman-Derr 2018). A greater abundance of Gram-positive bacteria (Actinobacteria and Firmicutes) than Gram-negative bacteria (Proteobacteria, Verrucomicrobia and Bacteroidetes) was observed under the drought stress condition (Acosta-Martinez et al. 2014). *RR* genotype could provide an added advantage in conferring drought tolerance and higher yield especially under the water-deficit condition as it naturally enriches Gram-positive bacteria in its microbiome, which confers drought tolerance without any seed treatment with beneficial microbes. In contrast to bacterial diversity and abundance in the rhizospheres, there was no variation in the fungal diversity and abundance between well-watered and water-deficit conditions of both genotypes in the current study. This is consistent with the earlier reports that the destabilizing effect of drought can be more evident in the bacterial community shift relative to the fungal community shift (de Vries et al. 2018).

Overall, the enrichment of Actinobacteria, Alphaproteobacteria (well-watered *RR* rhizosphere) and Firmicutes (water-deficit *RR* rhizosphere) in the rhizosphere of *RR* mutant indicated that the secreted root exudates might have the potential to select these phyla against other bacterial phyla. These enriched phyla belong to root growth-promoting bacteria with additional functions of drought tolerance and disease suppression (Yandigeri et al. 2012; Selim et al. 2019). Comparative field studies for biomass and seed yield differences between BTx623 and purified *RR* mutant (at least five times backcrossed) will reveal the real potential of *RR* for varietal and hybrid seed production. Further, multi-location trials across the sorghum growing regions of US in collaboration with plant breeders will provide comprehensive data as the soil type and microbiome composition will vary in different locations. Since sorghum is a monocot species similar to rice, maize and switch grass, the benefits of *RR* mutation, once proven, can be utilized for mechanistic studies as well as practical application in other monocot species. Further, identification of the gene/locus of the *RR* mutant will reveal the mechanism involved in the induced root secretion system, signaling mechanism, gene regulatory networks involved and metabolic pathways.

### *Author contribution statement*

VM conceived the idea. ZX provided the screening population. VB, LD, CC and NY performed the experiments. VB and LD performed the microbiome analysis. VB, LD, NY, CC, ZX and VM wrote the manuscript.

## Supplementary Information

Below is the link to the electronic supplementary material.

Supplementary file1 (PDF 4,553 KB)

Supplementary file2 (XLSX 33 KB)

Supplementary file3 (XLSX 55 KB)

Supplementary file4 (XLSX 3,158 KB)

## Data Availability

The raw reads of microbiome sequencing was submitted to NCBI SRA and can be accessed using BioProject ID: PRJNA660314 and BioSample accessions SAMN15945252, SAMN15945253.

## Abbreviations

*EMS*: Ethyl methanesulfonate
*OTU*: Operational taxonic unit
*RR*: Red root
