# Supplementary material for: Induced secretion system mutation alters rhizosphere bacterial composition in Sorghum bicolor (L.) Moench
Source: Planta. 2021 Jan 18;253(2):33. doi: 10.1007/s00425-021-03569-5 (PMC7813745; doi:10.1007/s00425-021-03569-5)

Article title: Induced secretion system mutant alters rhizosphere bacterial composition in *Sorghum bicolor (L.)* Moench.

Journal name: Planta

Authors: Vimal Kumar Balasubramanian, Lavanya Dampanaboina, Chris Cobos, Ning Yuan, Zhanguo Xin and Venugopal Mendu

a.

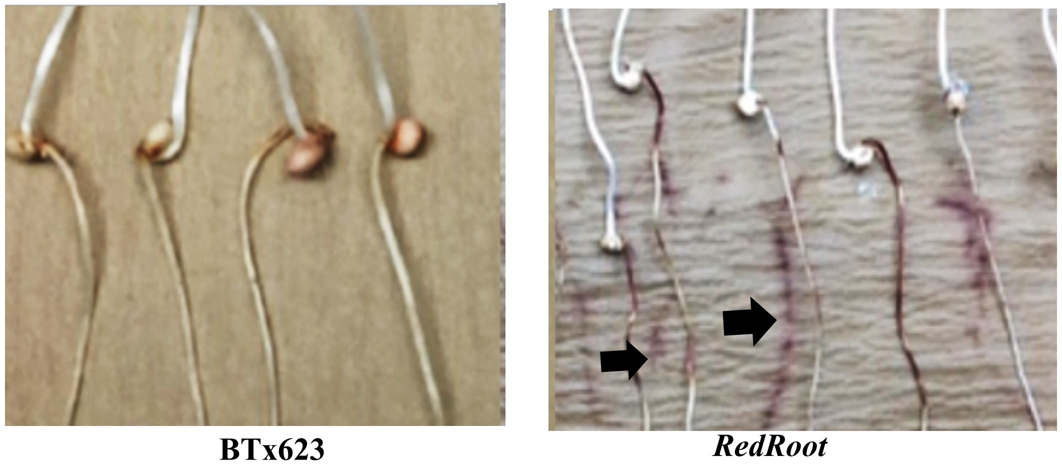

b.

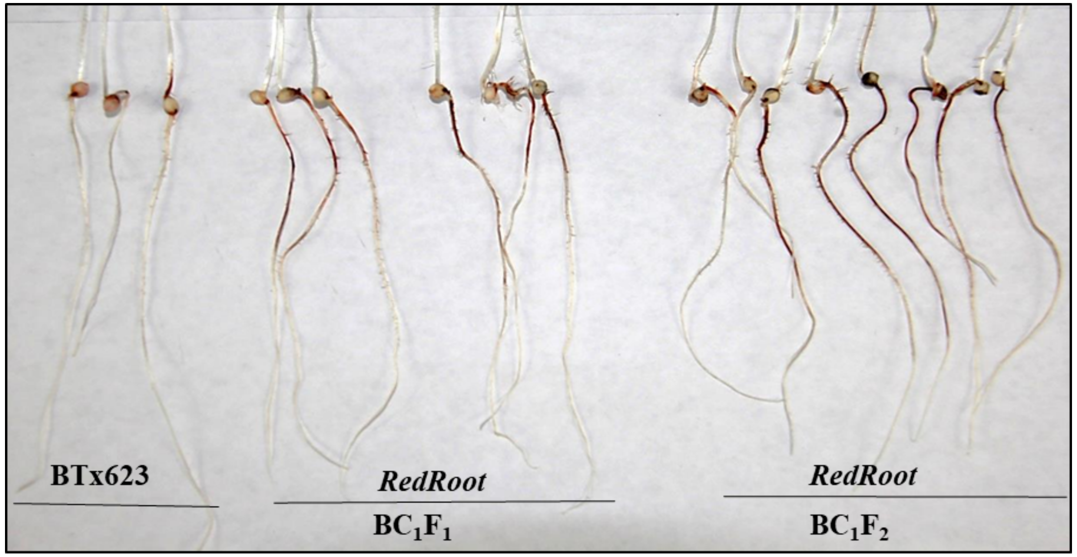

c.

**Chi-square probability test**				
Expected (E)	Observed (O)	Deviation (O-E)	$(O-E)^2$	$(O-E)^2/E$
147	143	-4	16	0.10884354
49	53	4	16	0.32653061
				0.43537415
			Degree of freedom	1

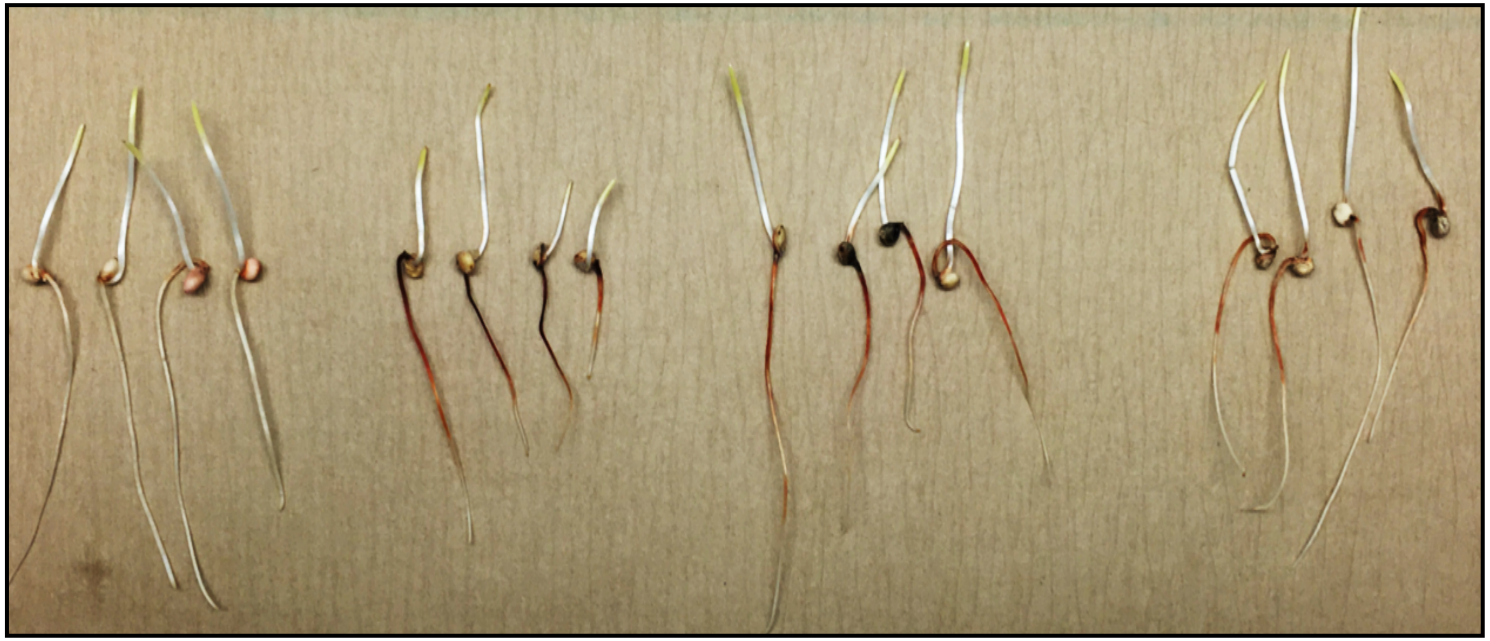
BTx623
Dark RedRoots
Medium RedRoots
Pale RedRoots

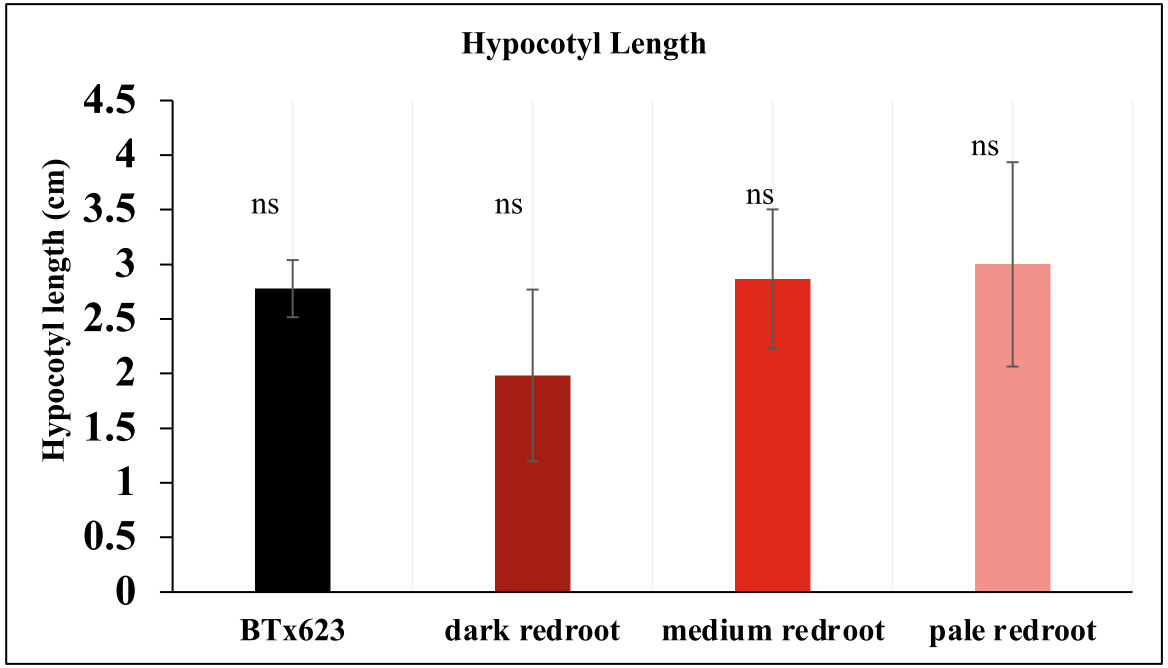
Hypocotyl Length
Hypocotyl length (cm)
4.5
4
3.5
3
2.5
2
1.5
1
0.5
0
ns
ns
ns
ns
BTx623
dark redroot
medium redroot
pale redroot

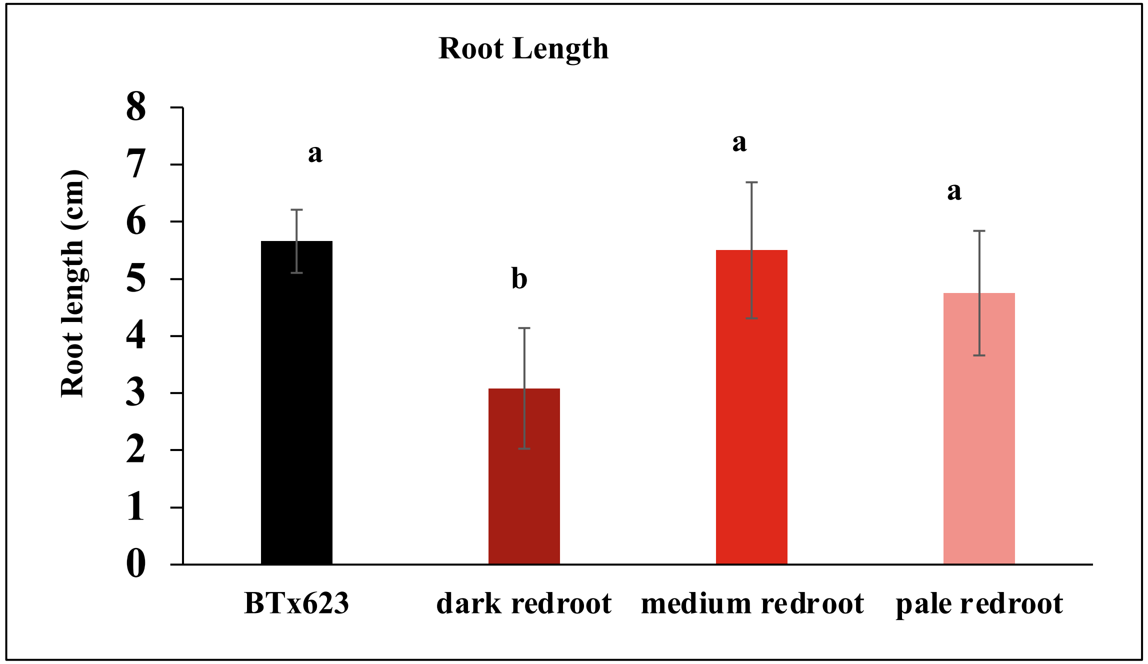
Root Length
Root length (cm)
8
7
6
5
4
3
2
1
0
a
b
a
a
BTx623
dark redroot
medium redroot
pale redroot

BTx623 (well-watered) | **BTx623 (water deficit)** | **RedRoot (well-watered)** | **RedRoot (water deficit)**

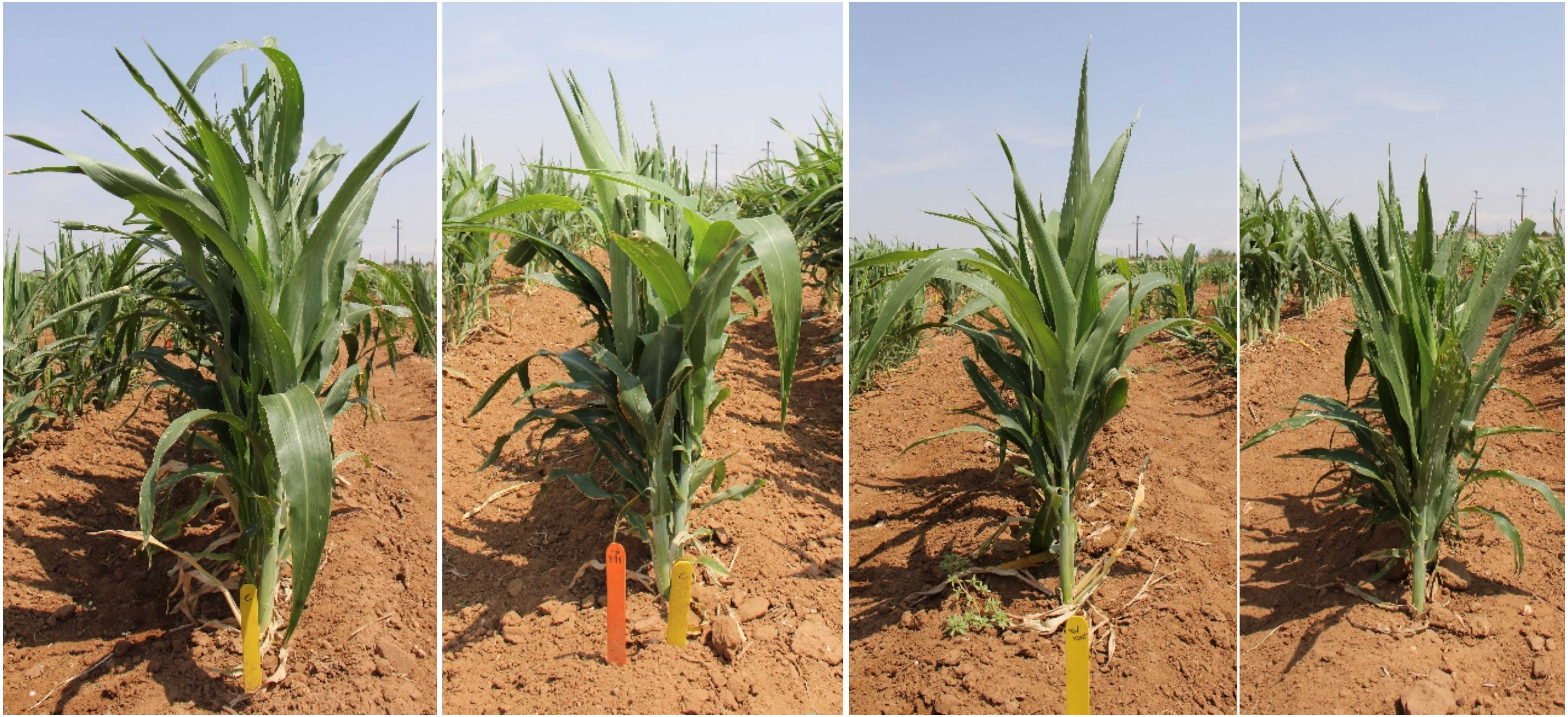

Bacteria – Phyla abundance

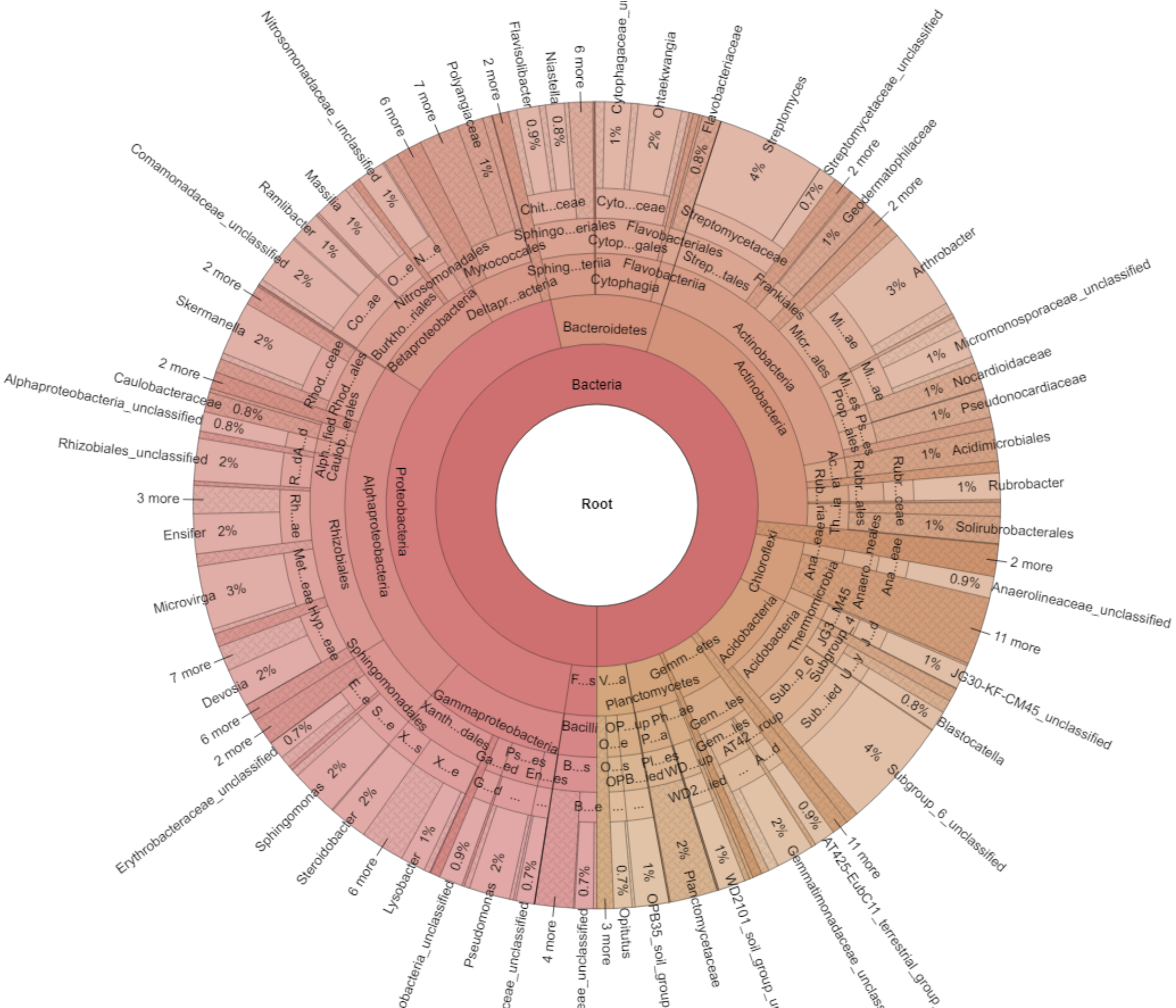

Fungi – Phyla abundance

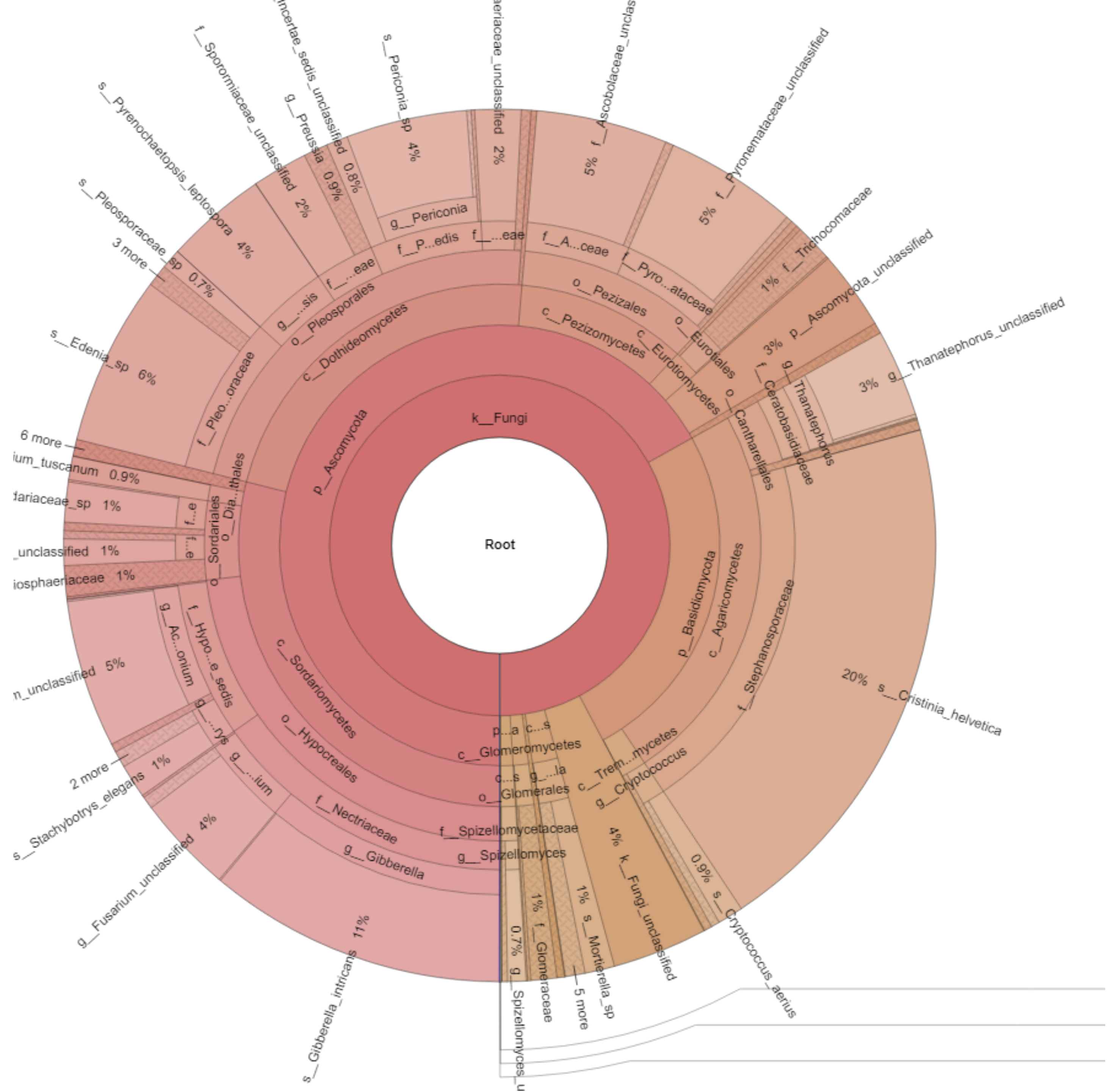

Supplement: Supplementary file 1 — Supplementary file1 (PDF 4,553 KB) [file 425_2021_3569_MOESM1_ESM.pdf]
